# A Systematic Review of Interventions—Does Invisalign Move Teeth as Effectively as Orthodontic Fixed Appliances?

**DOI:** 10.1155/sci5/4268902

**Published:** 2024-11-20

**Authors:** Mohammad Khursheed Alam, Mohammed Awawdeh, Nora Alhazmi, Khalid A. Abalkhail, Kiran Iyer, Huda Abutayyem, Haytham Jamil Alswairki, Mohammad Younis Hajeer

**Affiliations:** ^1^Orthodontic Division, Preventive Dentistry Department, College of Dentistry, Jouf University, Sakaka 72345, Saudi Arabia; ^2^Department of Dental Research Cell, Saveetha Dental College and Hospitals, Saveetha Institute of Medical and Technical Sciences, Chennai 600077, India; ^3^Department of Public Health, Faculty of Allied Health Sciences, Daffodil International University, Dhaka 1207, Bangladesh; ^4^Preventive Dental Science Department, College of Dentistry, King Saud Bin Abdulaziz University for Health Sciences (KSAU-HS), Riyadh 11426, Saudi Arabia; ^5^Preventive Dental Science Department, King Abdullah International Medical Research Center, Ministry of National Guard Health Affairs, Riyadh 11481, Saudi Arabia; ^6^Preventive Dental Science Department, Dental Services King Abdulaziz Medical City, Ministry of the National Guard-Health Affairs, Riyadh 11426, Saudi Arabia; ^7^Preventive Dental Science Department, College of Medicine & Dentistry, Ulster University, Birmingham B4 6BN, UK; ^8^Orthodontics Department, Department of Clinical Sciences, Center of Medical and Bio-Allied Health Sciences Research, College of Dentistry, Ajman University, P.O. Box 346, Ajman, UAE; ^9^School of Dental Sciences, Universiti Sains Malaysia, Kota Bharu 16150, Malaysia; ^10^Department of Orthodontics, Faculty of Dentistry, University of Damascus, P.O. Box 16046, Damascus, Syria

**Keywords:** brackets, fixed appliances, Invisalign, tooth movement

## Abstract

**Background:** Despite the large number of studies that have been done in this area, there is still a gap in the literature when it comes to comparing the orthodontic tooth movement (OTM) efficacy of Invisalign and fixed orthodontic appliances. The primary objectives of this study were to evaluate and compare the efficacy of Invisalign and fixed orthodontic appliances in terms of the amount and rate of OTM. Specifically, the study aimed to determine if there was a statistically significant difference between these two treatment modalities in achieving OTM and to assess whether treatment duration differs significantly between Invisalign and traditional fixed appliances. This investigation seeks to address the existing gaps in the literature by providing a clear comparison based on recent empirical evidence, thereby contributing to more informed treatment decisions in orthodontic practices.

**Methodology:** Relevant MeSH keywords and Boolean operators were selected by a team of reviewers to search several online databases for papers that were in accordance with the objectives of our review.

**Results:** At the end of the search protocol, 10 studies were deemed to be eligible for inclusion in the review. The pooled analysis revealed a statistically significant reduction in treatment time for patients using Invisalign compared to those with fixed appliances, with a total OR of 0.61 [95% CI 0.43, 0.85]. No significant heterogeneity was detected (*I*^2^ = 0%), and the test for overall effect was significant (*Z* = 2.86, *p*=0.004). Furthermore, a nonsignificant trend favoring Invisalign was shown, with an odds ratio of 1.43 and a confidence interval that included 1 (0.97, 2.10). The *p* value was 0.07, and there was negligible heterogeneity among studies, as indicated by an *I*^2^ of 0%.

**Conclusion:** Based on the findings from the selected studies, it can be concluded that Invisalign and fixed orthodontic appliances have similar overall efficacy in eliciting OTM. However, Invisalign treatment requires significantly less time to complete than fixed orthodontic appliances. Despite these observations, further studies are required to explore the long-term stability of OTM achieved with Invisalign and fixed orthodontic appliances.

**Registration and Protocol:** Registration was done in accordance with the Preferred Reporting Items for Systematic Reviews and Meta-Analyses (PRISMA) standards (CRD42023405593). The research protocol was created to meet the goals and was properly filed with PROSPERO; however, it has not been prospectively registered.

## 1. Introduction

Orthodontic treatment can result in various outcomes, which may include changes in tooth position, jaw growth and development, occlusion, esthetics, function, and oral health [1]. The primary goal of orthodontic treatment is to achieve a stable, functional, and esthetically pleasing occlusion, which can improve oral health and quality of life [2].

Orthodontic tooth movement (OTM) is a critical factor in achieving orthodontic treatment outcomes related to tooth position, occlusion, and esthetics [1, 2]. The amount and direction of tooth movement depend on the applied forces and the inherent properties of the tooth and surrounding bone. Therefore, orthodontic practitioners must carefully plan and execute treatment to achieve the desired tooth movement while minimizing potential side effects, such as root resorption, gingival recession, and relapse [3]. OTM can also influence jaw growth and development, particularly in adolescent patients [4]. The biological underpinnings of OTM are rooted in the remodeling capabilities of the periodontal ligament and alveolar bone. The application of orthodontic forces initiates a cascade of cellular and molecular responses, characterized by osteoclast-mediated bone resorption on the pressure side and osteoblast-driven bone formation on the tension side [4]. This bone remodeling is a critical component of OTM and is also implicated in adaptive alterations in the jaw's morphology. Orthodontic forces applied to the maxilla and mandible can affect the growth and remodeling of the bones, leading to changes in facial esthetics and occlusion [4].

Advancements in the domain of orthodontic aligner systems have been propelled by escalating patient demand for esthetically pleasing, comfortable, and hygienic treatment options. These systems have undergone significant evolution, with innovations in the material science and fabrication processes of clear orthodontic devices [5–11]. The progressive enhancement of these technologies has broadened the spectrum of malocclusions amenable to treatment via clear aligner therapy [10]. Clear aligner therapy offers several advantages including esthetic appeal, enhanced comfort, superior facilitation of dental hygiene, and a reduction in the intensity of discomfort relative to fixed orthodontic devices. Additionally, this modality has been associated with a decreased frequency and duration of clinical appointments as well as a diminished necessity for unscheduled emergency interventions [10]. Nonetheless, the utilization of clear aligners is constrained by higher production costs, the imperative for patient adherence, and limitations in addressing certain complex malocclusive states [11].

In addition to tooth position and jaw growth, orthodontic treatment can also impact occlusion, which refers to the way the teeth come together during biting and chewing. Proper occlusion can improve oral health, reduce the risk of dental problems, and enhance overall function [1]. OTM plays a crucial role in achieving optimal occlusion, and orthodontic treatment planning must consider the patient's bite relationship and the desired occlusal outcome [2]. Moreover, orthodontic treatment can impact esthetics, which refers to the appearance of the teeth and face [3]. The correct positioning of teeth can enhance facial symmetry, reduce crowding, and improve the overall appearance of the smile [4].

Studies have shown that clear aligners can effectively move teeth, with similar efficacy to fixed appliances. One of the studies found that clear aligners were as effective as fixed appliances in achieving tooth movement [5]. However, it should be noted that clear aligners may have some limitations in terms of the types of tooth movements that can be achieved. Another study found that clear aligners were less effective than fixed appliances in achieving rotational movements of teeth [6]. Additionally, clear aligners may require more frequent changes and patient compliance in terms of wearing them for the recommended amount of time each day [6].

During the alignment stage, self-ligating brackets have been found to dramatically improve dental irregularity more than conventional brackets; however, the bracket type at this stage had no effect on pain [7]. It was reported that patients treated with ceramic brackets reported more severe pain for a longer period of time than those treated with conventional brackets [8]. The study found that there was no significant difference in the rate of OTM between ceramic and metal brackets (0.66 mm/month vs. 0.64 mm/month). However, the study did note that ceramic brackets may be more prone to failure compared to metal brackets. Another study by Eberting et al. [9] compared the rate of OTM between different types of orthodontic brackets, including conventional metal brackets, ceramic brackets, and a low-friction self-ligating bracket system. The study found that the low-friction self-ligating bracket system was associated with a significantly greater rate of OTM compared to conventional metal and ceramic brackets (0.93 mm/month vs. 0.75 mm/month and 0.59 mm/month, respectively).

However, despite the extensive number of studies conducted in this subject area, there is still a gap in the literature when it comes to the comparison between Invisalign and fixed orthodontic appliances in terms of their OTM efficacy. While some studies have shown that Invisalign is comparable to fixed appliances in terms of the amount and rate of tooth movement [11], other studies have reported conflicting results [12]. Additionally, most of the studies conducted so far have been short-term and have small sample sizes, limiting their ability to draw definitive conclusions about the long-term effectiveness of Invisalign compared to fixed appliances [11–13]. Moreover, there is also a lack of standardization in the methodology used to evaluate OTM, making it difficult to compare results across studies. Therefore, the primary objectives of this investigation were to compare the effectiveness of Invisalign and fixed orthodontic appliances in terms of OTM and determine if there was a statistically significant difference between the two treatment modalities in terms of the amount and rate of OTM achieved. The secondary objectives were to evaluate the quality of the evidence available for this comparison, assess for any publication bias, and explore potential sources of heterogeneity across studies.

## 2. Materials and Methods

### 2.1. Review Protocol

**Figure 1:**
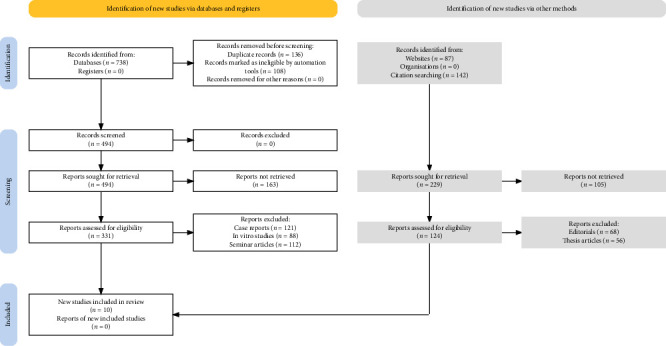
PRISMA strategy implementation for selection of studies in this review.

The PRISMA guidelines [14] were followed to conduct this study and ensure that this systematic review and meta-analysis were handled in a transparent and reproducible manner.  Figure 1 shows the study selection process for this review utilizing these guidelines.  Table 1 shows the PICOS protocol that was devised to identify the relevant population, intervention, comparison, outcome, and study design pertaining to the objectives of this review.

### 2.2. Search Protocol Across Databases

PubMed, MEDLINE, Scopus, Google Scholar, Cochrane Library, and Embase were searched for studies to include in this systematic review and meta-analysis. In each of these databases, we used a combination of MeSH keywords and Boolean operators (“AND” and “OR”) to make sure we did not miss any relevant studies, the results of which have been elucidated through Table 2.

### 2.3. Criteria for Inclusion and Exclusion

The inclusion and exclusion criteria that were defined to identify relevant studies for inclusion in this review have been shown through Table 3 in terms of the PICOS protocol.

### 2.4. Study Selection and Data Extraction

Two independent reviewers, both trained orthodontic experts with substantial experience in the domain of orthodontics, were tasked with the evaluation of study eligibility and the subsequent data extraction from the accepted studies. Their expertise extended to both Invisalign and fixed orthodontic appliances, ensuring a proficient assessment of the relevant literature. During the process, standardized data extraction forms were utilized to methodically record pertinent details, such as study and participant characteristics, interventions, outcomes, and results. To mitigate any potential for bias and to verify the precision and thoroughness of the collected data, the reviewers engaged in a meticulous cross-checking procedure. Any instances of disagreement or discrepancy in the data extraction process were addressed through a consultative approach, wherein a third expert reviewer was called upon to mediate and facilitate a consensus.

### 2.5. Evaluation of Bias

**Figure 2:**
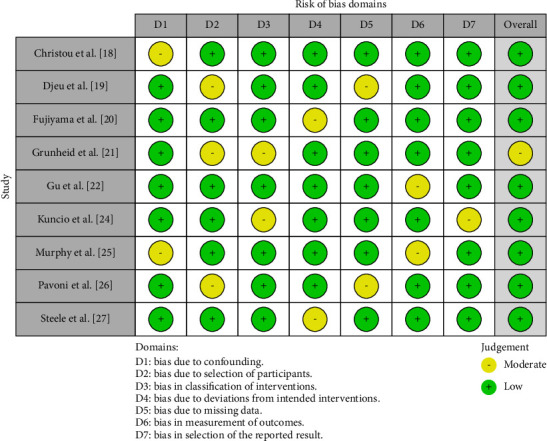
Risk of bias assessment in the selected papers using ROBINS-I.

**Figure 3:**
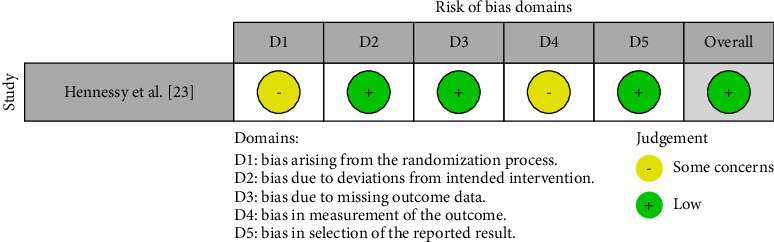
Risk of bias assessment in the selected papers using RoB 2.0.

To assess the quality of the included studies and the risk of bias, the ROBINS-I [15] tool (Figure 2) was utilized for the nonrandomized studies, the case-control and cohort papers that were to be included in this review, whereas Cochrane's risk of bias 2.0 tool (RoB 2.0) [16] was employed (Figure 3) for assessment of bias in the randomized controlled trials (RCTs) to be considered for inclusion in this review.

### 2.6. Meta-Analysis Protocol

RevMan 5 software was used to conduct the meta-analysis in this systematic review. The software provides a platform for the statistical analysis of data from the included studies. The meta-analysis was conducted using a fixed-effects (FE) model, assuming a 95% CI. Forest plots were generated for each outcome of interest using the OR as the effect size. The OR represents the odds of an event occurring in the intervention group relative to the control group. The FE model was chosen based on the assumption of homogeneity across studies, and that the included studies would be estimating the same underlying treatment effect. The results of the meta-analysis were interpreted based on the statistical significance and the magnitude of the effect size. RevMan 5 provided a robust platform for the statistical analysis of the data from the included studies, allowing for a more comprehensive and reliable evaluation of the effectiveness of Invisalign compared to fixed orthodontic appliances.

### 2.7. Certainty Assessment

The GRADE certainty assessment [17] for the studies included in the review was conducted retrospectively, with careful consideration of various parameters. The assessment encompassed study design, the number of studies, observed common findings, risk of bias, inconsistency, indirectness, imprecision, and other potential factors influencing certainty, in conjunction with the NOS and RoB 2.0 tool.

## 3. Results

### 3.1. Study Selection Process

As shown through Figure 1, the study selection process for this systematic review was conducted in a series of structured phases. Initially, new studies were identified through databases and registers, yielding 738 records from databases and none from registers. Prior to screening, a total of 136 duplicate records were removed, along with 108 records that were marked as ineligible by automation tools, leaving no records removed for other unspecified reasons. Further identification of potential studies was achieved through alternative sources: 87 records were found via websites and an additional 142 records were identified through citation searching. This resulted in 494 records being screened for relevance. All 494 records initially screened were then sought for retrieval. However, of these, 163 reports could not be retrieved, which necessitated seeking 229 reports through other methods. Subsequently, 331 reports were assessed for eligibility. This assessment led to the exclusion of 121 case reports, 88 in vitro studies, and 112 seminar articles, which were deemed unsuitable for inclusion in the review. A further 124 reports were assessed, from which 68 editorials and 56 thesis articles were excluded. At the conclusion of the selection process, 10 studies [18–27] met the inclusion criteria and were incorporated into the review.

### 3.2. Bias Assessment Results

For Christou et al. [18], the bias due to confounding (D1) was moderate, but the study was determined to have a low risk of bias across all other domains (D2–D7), leading to an overall low risk of bias. Djeu et al. [19] exhibited low risk of bias in the domains of bias due to selection of participants (D2), classification of interventions (D3), deviations from intended interventions (D4), and measurement of outcomes (D6), with moderate concerns in the domain of bias due to missing data (D5), resulting in an overall low risk of bias. Fujiyama et al. [20] were judged as having low risk across most domains, except for a moderate risk in bias due to deviations from intended interventions (D4), maintaining an overall low risk of bias. Grunheid et al. [21] had low risk in most domains, though they had moderate risk in bias due to selection of participants (D2) and classification of interventions (D3), which led to an overall moderate risk of bias. Gu et al. [22] showed a low risk of bias in nearly all domains but had moderate concerns in bias in measurement of outcomes (D6), with an overall low risk of bias. Kuncio et al. [24] had low risk assessments in most domains, with moderate concerns in bias in classification of interventions (D3) and bias in selection of the reported result (D7), resulting in an overall low risk of bias. Murphy et al. [25] presented a moderate risk in bias due to confounding (D1) and measurement of outcomes (D6) but maintained a low risk across other domains, leading to an overall low risk of bias. Pavoni et al. [26] showed a low risk of bias in most domains, while having moderate concerns in bias due to selection of participants (D2) and bias due to missing data (D5), resulting in an overall low risk of bias. Steele et al. [27] were assessed as having low risk across all domains, except for a moderate risk in bias due to deviations from intended interventions (D4), culminating in an overall low risk of bias. Hennessy et al. [23] exhibited “some concerns” in the bias arising from the randomization process (D1) and in bias due to deviations from intended intervention (D4). It was found to have a low risk of bias in other domains, including bias due to missing outcome data (D3), bias in measurement of the outcome (D4), and bias in selection of the reported result (D5), leading to an overall judgment of “low risk” of bias.

### 3.3. GRADE Assessment Results

Table 4 shows the GRADE assessment conducted for the studies that were included in the review. For the three case-control studies included, it was observed that there were no discernible differences in pretreatment or posttreatment orthodontic measurements [18, 21, 25]. The risk of bias in these studies was determined to be low. There was also a low degree of inconsistency and indirectness among the findings, and the imprecision was deemed low. Given these factors, the certainty of the evidence from the case-control studies was rated as moderate. Three comparative cohort studies indicated that patients in the Invisalign group completed their treatment faster, yet consistently scored lower in certain orthodontic measurements compared to those with fixed appliances [19, 24, 26]. The risk of bias for these studies ranged from low to moderate, while inconsistency and indirectness were found to be low. The low level of imprecision resulted in a moderate certainty of evidence. The retrospective cohort studies, also numbering three, showed similar effectiveness in treating certain orthodontic conditions, with Invisalign being marginally more effective in cases of deep overbite [20, 22, 27]. The risk of bias was assessed as low to moderate, with low inconsistency and indirectness, and low imprecision in the findings. The certainty of evidence for these studies was thus considered moderate. The single RCT [23] that demonstrated comparable effectiveness between invisalign and fixed appliances in term of mandibular incisor proclination had a low risk of bias. The RCT had a low risk of bias. Since it was a single study, inconsistency was not applicable. Indirectness and imprecision were both low, and no other factors were reported that would diminish the certainty of the evidence. Consequently, the certainty of evidence from the RCT was rated as high.

### 3.4. Demographic Characteristics of the Included Studies

Table 5 provides information on the 10 studies [18–27] that were selected for this review in terms of their demographic characteristics. The studies have been conducted in the USA, Japan, Ireland, and Italy, with sample sizes ranging from 22 to 96 participants. The age range of the participants varies from 12 to 64 years. It is important to note that some studies have unspecified values for the age range, making it difficult to draw comparisons across the studies. The gender ratio across the studies is predominantly female, with the highest female-to-male ratio being reported in the study by Gu et al. [22], with 62 females out of a sample size of 96. Similarly, the study by Grunheid et al. [21] also reported a high female-to-male ratio, with 44 females out of a sample size of 60. However, the study by Djeu et al. [19] did not report the gender ratio of their sample. Overall, the table suggests that there is a gender bias in dental research, with female participants being overrepresented in the studies. This could have implications for the generalizability of the findings, as dental health and treatment outcomes may differ between males and females.

### 3.5. Assessed Parameters

Table 6 presents the associated inferences and parameters that were utilized across the selected trials. Christou et al. [18] conducted a case-control study where the Invisalign group completed treatment in 19.52 months, which was notably quicker than the 23.62 months for the group with conventional fixed appliances prepared using the Edgewise technique with 0.22-inch slots. The study involved 29 participants for each treatment group. Djeu et al. [19] reported on a comparative cohort study in which braces were employed as the fixed appliance. The treatment duration for the Invisalign group was approximately 1.4 years, compared to 1.7 years for the fixed appliance group, with 24 participants in each cohort. Fujiyama et al. [20] presented a retrospective cohort study without specifying the type of fixed appliance or the mean treatment period. The groups were evenly matched with 25 participants undergoing treatment with Invisalign and 25 with the conventional appliance. Grunheid et al. [21] utilized a case-control study design where both groups were treated with appliances prepared using the Edgewise technique with 0.22-inch slots. The Invisalign group concluded their treatment in 13.4 months, whereas the fixed appliance group finished in 20.2 months. Each group included 30 individuals. Gu et al. [22] examined a retrospective case-control cohort without specifying the type of fixed appliance used. The Invisalign group's treatment lasted 13.35 months, in stark contrast to the 19.08 months observed for the fixed appliance group. Each group consisted of 48 participants.

Hennessy et al. [23] designed a RCT using self-ligating brackets with 0.22-inch slots prepared with the Edgewise technique. The treatment periods were 10.2 months for the Invisalign group and 11.3 years for the fixed appliance group, each comprising 22 participants. Kuncio et al. [24] reported on a comparative cohort study where braces were the fixed appliance of choice. The Invisalign group underwent treatment for 1.74 years, while the fixed appliance group took 2.34 years. There were 11 participants in each treatment group. Murphy et al. [25] conducted a case-control study that did not specify the treatment duration. Both the Invisalign group and the fixed appliance group, which used braces, included 30 participants each. Pavoni et al. [26] provided data from a comparative cohort study with self-ligating brackets as the fixed appliance. All groups underwent treatment for an average duration of 1.8 years, with each group comprising 20 individuals. Steele et al. [27] conducted a retrospective cohort study with miniplates placed at the first molar position as the fixed appliance. The Invisalign group completed their treatment in 19.75 months, while the fixed appliance group required 29.39 months. The study included 29 participants in the Invisalign group and 24 in the fixed appliance group.

### 3.6. Efficacy Comparison Between Invisalign and Fixed Appliances in Eliciting OTM

**Figure 4:**
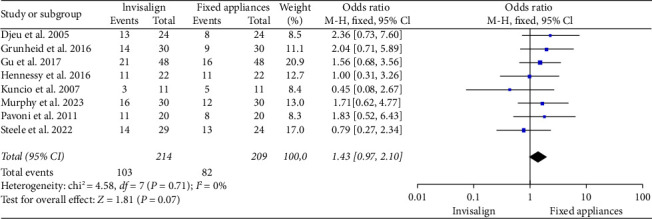
OR representation of the efficacy comparison between Invisalign and fixed appliances in eliciting OTM.

The forest plot in Figure 4 synthesized data from the included papers studies to compare the efficacy of Invisalign with fixed appliances in eliciting OTM. The pooled OR across all studies was 1.43 [0.97, 2.10], suggesting a trend that Invisalign may be more effective than fixed appliances in eliciting OTM. However, this effect did not reach conventional levels of statistical significance, as the confidence interval just included 1, and the *p* value was 0.07, indicating a 7% probability of observing such a difference (or more extreme) if there was no actual difference in efficacy. The heterogeneity of the included studies was assessed through a chi-squared test, yielding a value of 4.58 with 7 degrees of freedom (*p*=0.71), and an *I*^2^ of 0%, suggesting very low to no heterogeneity among the study results, meaning that the studies were sufficiently similar in their findings.

### 3.7. Mean Time of Completion of Treatment (in Months) of Invisalign and Fixed Orthodontic Appliances

**Figure 5:**
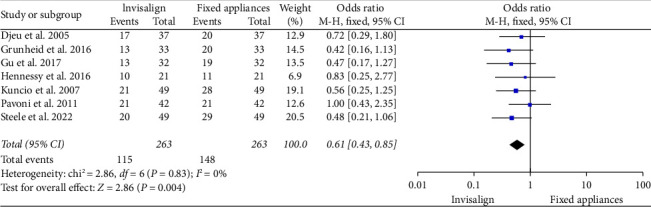
OR representation of the mean time of completion of treatment (in months) of Invisalign and fixed orthodontic appliances as observed in the selected studies.

The forest plot presented in Figure 5 illustrates a meta-analysis comparing the mean time of completion of treatment using Invisalign and fixed orthodontic appliances. The total pooled OR for all studies was 0.61 [95% CI 0.43, 0.85], suggesting that, on average, Invisalign could be associated with a shorter treatment duration compared to fixed appliances when considering all studies in the analysis. This pooled result was statistically significant, as indicated by the *Z*-test for overall effect (*Z* = 2.86, *p*=0.004), and did not show evidence of heterogeneity (chi^2^ = 2.86, degrees of freedom (*df*) = 6, *p*=0.83; *I*^2^ = 0%). The *I*^2^ value of 0% indicates no observed heterogeneity among the included studies, suggesting the effect estimates were consistent across studies.

## 4. Discussion

This study is significant as it provides insights into two different aspects of dental research: the representation of genders in dental research and a comparison between the efficacy of Invisalign and fixed appliances in orthodontic treatment. The findings of the study reveal that female participants are overrepresented in dental research, which may affect the generalizability of the results as dental health and treatment outcomes may differ between males and females. The comparison between the efficacy of Invisalign and fixed appliances in orthodontic treatment revealed that while both methods are effective in treating malocclusion, fixed appliances may be more effective in certain aspects, such as movement across the frontal plane, buccal corridors, gingival display, maxillary dental midline, and canine rotation and tipping. On the other hand, Invisalign was effective in maxillary incisor inclination and position and completed the treatment sooner than the fixed appliance group in some studies. The statistical analysis demonstrated that the overall efficacy of Invisalign and fixed appliances in eliciting OTM was similar, and the mean time of completion of treatment was shorter in the Invisalign group in some studies. In summary, this study highlights the need for a balanced representation of genders in dental research and provides valuable insights into the efficacy of Invisalign and fixed appliances in orthodontic treatment, which can guide treatment decisions for dental practitioners.

Christou et al. [18] discovered that traditional fixed appliances were more effective in managing aspects such as frontal plane movement, buccal corridors, gingival display, maxillary dental midline, and smile index. However, they found the Invisalign system to be effective in controlling maxillary incisor inclination and position, a finding that is echoed in Djeu et al.'s study [19], where Invisalign was associated with a lower buccolingual inclination, and Grunheid et al.'s study [21], where Invisalign resulted in a higher buccolingual inclination after treatment. Djeu et al. [19] and Gu et al. [22] both reported that Invisalign was associated with faster treatment completion times, a beneficial aspect that was not highlighted in the other studies. However, Djeu et al. [19] observed that Invisalign consistently scored lower than fixed appliances in terms of overjet, occlusal contacts, buccolingual inclination, and occlusal relationships, a finding that contrasts with Fujiyama et al. [20] who found Invisalign to be slightly more effective in treating severe deep overbite.

When it comes to the treatment of severe deep overbite, Fujiyama et al. [20] found both Invisalign and traditional fixed appliances to be effective but gave a slight advantage to Invisalign. This contrasts with the findings of other studies such as Djeu et al. [19] and Gu et al. [22], which found fixed appliances to be more effective in treating malocclusion and overjet. In terms of buccolingual inclination and canine orientation, Grunheid et al. [21] found the Invisalign group to have a higher inclination after treatment and a significant increase in intercanine distance. This contrasts with the findings of Murphy et al. [25], who reported more OTM of the maxillary teeth in all directions, especially in terms of rotation and tipping of the canine with fixed appliances. The degree of similarity among studies advocating for fixed appliances [19, 25, 26] was notable and presented a strong case for their comprehensive capabilities. In contrast, the studies supportive of Invisalign [18, 20, 27] highlighted niche areas where it may be preferable. The divergence in findings underscores the importance of individualized treatment planning, where the selection of the orthodontic modality must be aligned with the specific treatment goals and patient preferences.

As far as orthodontic literature is concerned, overall quality of life in patients (along with their esthetics) improves with clear aligners as compared to traditional fixed braces [28, 29]. Conversely, clear aligners also demonstrate certain limitations in terms of reining in tooth mobility [30]. However, fewer high-quality studies were found to support the therapeutic efficacy of transparent aligners compared to traditional appliances, leaving doctors to rely more on clinical judgment and raising the risk of adverse therapeutic effects. After doing a thorough search, Lagravere [13] was unable to locate any research testing the therapeutic effects of clear aligners. Following that, a recent comprehensive review found that clear aligners were successful in reducing anterior intrusion and posterior buccolingual inclination but not anterior buccolingual inclination [12]. The most challenging movement was found to be extrusion, which was followed by rotation in another systematic review that compared clear aligners with fixed orthodontic appliances [11]. The most predictable bodily distalization of the upper molar within 1.5 mm was found in this study. Thus, in cases of straightforward malocclusions, transparent aligners were advised [12]. Zeng et al. conducted a review in 2014 and only discovered one pertinent paper when comparing clear aligners and braces [4].

The buccal and coronal forces applied by braces to the center of resistance of teeth were demonstrated in a study by Isaacson et al. [31]. During alignment, this can cause tipping and proclination. Clear aligners can move one or more teeth at a time to straighten them independently, and the proclination of teeth might be reduced by this progressive, segmented movement. Clear aligners might be appropriate for patients with thin gingival biotypes in order to reduce the possibility of gingival recession. There was a difference between the findings of two studies included in our review [19, 20] regarding occlusal connections and overjet. Djeu attributed the statistically inferior scores of clear aligners to the comparatively ineffective root torque control [19], whereas Zheng et al. found no statistically significant difference between the two groups [5]. The difference was most likely caused by the fact that this study included extraction cases, whereas the prior study did not.

### 4.1. Limitations

Our investigation presented limitations in terms of sample sizes and age ranges of the participants. Some studies did not report the gender ratio, which made it difficult to draw comparisons across the studies. Moreover, the findings suggested a gender bias in dental research, with female participants being overrepresented in the studies. This could affect the generalizability of the findings as dental health and treatment outcomes may differ between males and females. The study also had limitations in comparing the efficacy of Invisalign and fixed orthodontic appliances, as the studies used different protocols, appliances, treatment periods, and assessment methods. The findings suggested that both Invisalign and fixed appliances are effective in treating malocclusion, but fixed appliances tend to perform better in certain aspects, such as movement across the frontal plane, buccal corridors, gingival display, maxillary dental midline, and canine rotation and tipping. However, the study could not compare other factors such as patient satisfaction, comfort, and treatment duration. Finally, the forest plots showed no significant difference in the overall efficacy and treatment duration between Invisalign and fixed orthodontic appliances. Nonetheless, further studies are necessary to confirm these findings and evaluate other factors affecting the efficacy of orthodontic appliances.

### 4.2. Recommendations Pertaining to Clinical Practice

Based on the synthesis of data from our assessed findings, Invisalign may present a trend toward more effective OTM compared to fixed appliances, although this effect was not statistically significant. Despite this, Invisalign was associated with a shorter average treatment duration. Clinicians might consider Invisalign as a viable option for patients prioritizing treatment time, as it completed treatment sooner than fixed appliances. In terms of specific tooth movements, fixed appliances demonstrated superior results in the movement across the frontal plane, buccal corridors, gingival display, maxillary dental midline, and smile index. In contrast, Invisalign showed effectiveness in maxillary incisor inclination and position. It is advisable to take into account the nature of the malocclusion and the desired tooth movements when selecting the appropriate orthodontic treatment modality. For patients with severe deep overbite, both Invisalign and fixed appliances were effective, with Invisalign being slightly more favorable. However, the Invisalign group showed more relapse than braces patients, suggesting that posttreatment retention strategies should be carefully considered with Invisalign. Considering these findings, orthodontic treatment should be tailored to individual patient needs, with an understanding of the relative strengths and limitations of each modality. Clinicians are encouraged to integrate these insights with their professional judgment and patient preferences when developing treatment plans.

The study encountered several limitations that could impact the validity and applicability of its findings, including variable sample sizes and a lack of diversity in participant demographics, which may limit the generalizability across different populations. Not all studies reported gender ratios, and an observed overrepresentation of female participants could skew results, affecting the broader applicability of findings, as dental health outcomes can vary between genders. Additionally, the heterogeneity in treatment protocols, appliance types, and assessment methods across studies complicates direct comparisons between Invisalign and fixed appliances. Furthermore, the focus was predominantly on specific orthodontic movements, with less attention to patient satisfaction, comfort, and overall treatment experience, which are crucial for a holistic assessment of treatment efficacy. Lastly, the studies included did not sufficiently explore long-term treatment stability and relapse rates, essential factors for evaluating the enduring success of orthodontic interventions. These limitations underscore the need for further, more comprehensive research to substantiate the comparative effectiveness of different orthodontic modalities.

## 5. Conclusion

Invisalign and fixed orthodontic appliances were compared for efficacy in terms of OTM and treatment time in this systematic review and meta-analysis. It is possible to draw the conclusion that Invisalign and fixed orthodontic appliances have comparable overall efficacy in inducing OTM based on the results from the chosen research. However, compared to fixed orthodontic appliances, Invisalign treatment takes a lot less time to complete. The study's conclusions offer insightful information on the effectiveness of various kinds of orthodontic equipment, which can help physicians choose the best course of action for their patients. To learn more about the long-term stability of OTM attained with Invisalign and fixed orthodontic products, more research is needed.

## Figures and Tables

**Table 1:** PICOS protocol utilized for this review.

PICOS component	Description
Population	Patients undergoing orthodontic treatment with either Invisalign or fixed orthodontic appliances
Intervention	Use of Invisalign for orthodontic treatment
Comparison	Patients undergoing orthodontic treatment with fixed appliances
Outcome	Effectiveness of Invisalign compared to fixed orthodontic appliances in terms of OTM
Study design	Randomized controlled trials, quasi-randomized controlled trials, and observational studies

**Table 2:** Search strings utilized across the different databases.

Database	Search string (MeSH keywords/free-text terms)	Boolean operators used	Search date range
PubMed	(“Orthodontics” [MeSH Terms] OR “Orthodontics” [All Fields]) AND (“Tooth Movement Techniques” [MeSH Terms] OR “Tooth Movement” [All Fields]) AND (“Invisalign” [MeSH Terms] OR “Invisalign” [All Fields]) AND (“Orthodontic Appliances, Removable” [MeSH Terms] OR “Removable Orthodontic Appliances” [All Fields])	“AND,” “OR”	January 2000–January 2023
MEDLINE	(“Orthodontics” [MeSH Terms] OR “Orthodontics” [All Fields]) AND (“Tooth Movement” [MeSH Terms] OR “Tooth Movement” [All Fields]) AND (“Invisalign” [MeSH Terms] OR “Invisalign” [All Fields]) and (“Orthodontic Appliances, Removable” [MeSH Terms] OR “Removable Orthodontic Appliances” [All Fields])	“AND,” “OR”	January 2000–January 2023
Scopus	(“Invisalign” OR “Aligners”) AND (“Orthodontic Appliances, Removable” OR “Removable Orthodontic Appliances”) AND (“Orthodontic Brackets” OR “Braces”) AND (“Tooth Movement” OR “Dental Movement”)	“AND,” “OR”	January 2000–January 2023
Google Scholar	(“Invisalign” OR “Clear Aligners”) AND (“Orthodontic Fixed Appliances” OR “Brackets”) AND (“Tooth Movement” OR “Dental Displacement”) AND “Effectiveness”	“AND,” “OR”	January 2000–January 2023
Cochrane Library	(“Invisalign” OR “Clear Aligners”) AND (“Orthodontic Brackets” OR “Fixed Orthodontic Appliances”) AND (“Orthodontic Appliances, Removable” OR “Removable Orthodontic Appliances”) AND (“tooth movement Techniques” OR “Tooth Movement”)	“AND,” “OR”	January 2000–January 2023
Embase	(“Orthodontics”/exp OR “Orthodontics”) AND (“Tooth Movement”/exp OR “Tooth Movement”) AND (“Invisalign”/exp OR “Invisalign”) AND (“Orthodontic Appliances, Removable”/exp OR “Removable Orthodontic Appliances”)	“AND,” “OR”	January 2000–January 2023

**Table 3:** Inclusion and exclusion criteria delineated for this review.

PICOS component	Description	Inclusion criteria	Exclusion criteria
Population	Patients undergoing orthodontic treatment with either Invisalign or fixed orthodontic appliances	Studies conducted on human subjects	Studies including patients with craniofacial anomalies
Intervention	Use of Invisalign for orthodontic treatment	Studies that compared the effectiveness of Invisalign and fixed orthodontic appliances in moving teeth	Other companies offering clear aligners
Comparison	Patients undergoing orthodontic treatment with fixed appliances	—	—
Outcome	Effectiveness of Invisalign compared to fixed orthodontic appliances in OTM	Studies that reported quantitative data on tooth movement outcomes such as the amount of tooth movement, treatment duration, and occlusal outcomes	Studies that did not include clear definitions of treatment outcomes
Study design	Randomized controlled trials (RCTs), quasi-randomized controlled trials, and observational studies	Studies that were published in peer-reviewed journals	Studies that were not published in English, published before 2000, did not include a control group, were conducted on animals, or were case reports, reviews, or editorials

**Table 4:** GRADE assessment conducted for the review.

Study design	Number of studies	Inference observed	Risk of bias	Inconsistency	Indirectness	Imprecision	Others	Certainty
Case-control	3	No discernible pretreatment or posttreatment differences in various orthodontic measurements	Low	Low	Low	Low	None	Moderate
Comparative cohort	3	Invisalign group finished treatment faster but had consistently lower scores in certain orthodontic measurements	Low to moderate	Low	Low	Low	None	Moderate
Retrospective cohort	3	Similar effectiveness in treating certain conditions, with Invisalign being marginally more effective in deep overbite cases	Low to moderate	Low	Low	Low	None	Moderate
RCT	1	Comparable mandibular incisor proclination between Invisalign and fixed appliances, with no significant statistical difference	Low	Not applicable	Low	Low	None	High

**Table 5:** Demographic variables pertaining to the papers that were selected for this review.

Author ID	Year	Region	Sample size (*n*)	Age range (in years)	Gender ratio
Christou et al. [18]	2020	USA	58	12–30	39 females
Djeu et al. [19]	2005	USA	48	33.6 and 22.7 (mean)	Unspecified
Fujiyama et al. [20]	2022	Japan	50	23.2 ± 7.5 (mean)	Unspecified
Grunheid et al. [21]	2016	USA	60	12.7–64	44 females
Gu et al. [22]	2017	USA	96	26.0 ± 9.7 and 22.1 ± 7.9 (mean)	62 females
Hennessy et al. [23]	2016	Ireland	44	29.1 ± 7.5 and 23.7 ± 7.0 (mean)	27 females
Kuncio et al. [24]	2007	USA	22	31.82 (mean)	20 females
Murphy et al. [25]	2023	USA	60	Unspecified	Unspecified
Pavoni et al. [26]	2011	Italy	40	18 ± 1.5	21 females
Steele et al. [27]	2022	Multicentre	53	18.4–59	35 females

**Table 6:** Description of selected papers and the type of appliances, comparison groups, and outcomes assessed.

Author ID	Protocol	Fixed appliance used	Treatment period (mean)	Groups	OTM assessment
Christou et al. [18]	Case-control	Appliance with 0.22-inch slots (prepared using Edgewise technique)	19.52 months (Invisalign), 23.62 months (fixed appliance)	2 (29 people in Invisalign and 29 in conventional fixed appliance)	Regarding movement across the frontal plane, buccal corridors, gingival display, maxillary dental midline, and smile index, the fixed appliance group fared better, with Invisalign being effective in maxillary incisor inclination and position. However, neither group's pretreatment nor posttreatment ratings showed a discernible difference
Djeu et al. [19]	Comparative cohort	Braces	1.4 years (Invisalign), 1.7 years (fixed appliance)	2 (24 people in Invisalign and 24 in conventional fixed appliance)	Initial overjet, occlusion, and buccal posterior crossbite were all adversely linked with the movements of the Invisalign group. Additionally, Invisalign scores for overjet, occlusal contacts, buccolingual inclination, and occlusal relationships were consistently lower than braces scores. The Invisalign group, however, finished their treatment 4 months sooner than the other group
Fujiyama et al. [20]	Retrospective cohort	Type unspecified	Unspecified	2 (25 people in Invisalign and 25 in conventional fixed appliance)	Patients with a severe deep overbite were successfully treated with both Invisalign and traditional fixed appliances; however, Invisalign therapy was marginally more effective in those with deep overbite
Grunheid et al. [21]	Case-control	Appliance with 0.22-inch slots (prepared using Edgewise technique)	13.4 months (Invisalign), 20.2 months (fixed appliance)	2 (30 people in Invisalign and 30 in conventional fixed appliance)	Although there was no difference between the groups before treatment, the Invisalign group's buccolingual inclination was noticeably higher after treatment. With treatment, the canines in the fixed appliance group became more upright, whereas the buccolingual inclination did not significantly change in the Invisalign group. The intercanine distance, in contrast to the other group, rose dramatically over the course of therapy in the Invisalign group
Gu et al. [22]	Retrospective case-control	Type unspecified	13.35 months (Invisalign), 19.08 years (fixed appliance)	2 (48 people in Invisalign and 48 in conventional fixed appliance)	Similar OTM levels were seen in both groups, but the fixed appliance group showed somewhat superior efficacy in treating malocclusion. However, the Invisalign group completed their therapy 5.7 months earlier than the other group
Hennessy et al. [23]	RCT	Self-ligating brackets with 0.22-inch slots (prepared using Edgewise technique)	10.2 months (Invisalign), 11.3 years (fixed appliance)	2 (22 people in Invisalign and 22 in conventional fixed appliance)	Both groups produced roughly the same amount of mandibular incisor proclination, and there was no discernible statistical difference in their efficacy
Kuncio et al. [24]	Comparative cohort	Braces	1.74 years (Invisalign), 2.34 years (fixed appliance)	2 (11 people in Invisalign and 11 in conventional fixed appliance)	Both groups experienced significant shifts in mandibular anterior alignment and overall alignment, but only the Invisalign group experienced shifts in maxillary anterior alignment. However, Invisalign patients relapsed more frequently than braces patients
Murphy et al. [25]	Case-control	Braces	Unspecified	2 (30 people in Invisalign and 30 in conventional fixed appliance)	Patients with fixed appliances exhibited much more OTM of the maxillary teeth in all directions, especially in terms of rotation and tipping of the canine
Pavoni et al. [26]	Comparative cohort	Self-ligating brackets	1.8 years for all the groups	2 (20 people in Invisalign and 20 in conventional fixed appliance)	In comparison to the Invisalign group, the self-ligating group showed statistically significant differences in the transverse dentoalveolar width and the perimeter of the maxillary arch during treatment, with no difference in the overall treatment times between the two groups
Steele et al. [27]	Retrospective cohort	Miniplate (placed at the 1^st^ molar position)	19.75 months (Invisalign), 29.39 months (fixed appliance)	2 (29 people in Invisalign and 24 in fixed appliance)	Invisalign used maxillary and mandibular incisor extrusion, whereas fixed appliances did so via molar intrusion and anticlockwise mandibular autorotation

## Data Availability

All data are available within the article.

## Supporting Information

PRISMA 2020 checklist.
